# Supplementary material for: Evolution of Recrystallized Grain and Texture of Cold-Drawn Pure Mg Wire and Their Effect on Mechanical Properties
Source: Materials (Basel). 2020 Jan 16;13(2):427. doi: 10.3390/ma13020427 (PMC7014322; doi:10.3390/ma13020427)
Supplement: Supplementary file 1 [file materials-13-00427-s001.pdf]

materials

Evolution of recrystallized grain and texture of cold-drawn pure Mg wire and their effect on mechanical properties

Jiao Meng [1,2,a], Liuxia Sun [3,a], Yue Zhang [1,2], Feng Xue [1,2,4], Chenglin Chu [1,2] and Jing Bai [1,2,4,*]

[1] School of Materials Science and Engineering, Southeast University, Nanjing 211189, China; joejoe1st@yeah.net (J.M.); zy_seu@foxmail.com (Y.Z.); xuefeng@seu.edu.cn (F.X.); clchu@seu.edu.cn (C.C.)
[2] Jiangsu Key Laboratory for Advanced Metallic Materials, Southeast University, Nanjing 211189, China
[3] College of Arts and Sciences, Shanghai Dianji University, Shanghai 201306, China; spring3616@126.com
[4] Suzhou Institute of Biomedical Devices, Southeast University, Jiangsu 215000, China
[a] Jiao Meng and Liuxia Sun made equal contribution to this work
* Correspondence: baijing@seu.edu.cn; Tel.: +86 25 52090689

Received: 16 December 2019; Accepted: 14 January 2020; Published: date

Supplementary Figures

The tensile test was carried out on the Mg wires with the diameter of 1.5 mm. Figure S1 shows the strain-stress curves of Mg wires with different annealing treatments. The wires annealed at 150 °C and 200 °C showed poor plasticity, although the yield strength were relatively high. The plasticity was obviously improved after annealed at 250 °C. However, with the holding time prolonged to 120 min, there was an evident decline of yield strength. After annealed at 300 °C for 30 min, the wires obtained best plasticity, with no significant decrease of yield strength. With the further increase of temperature, both the plasticity and yield strength of Mg wires decreased.

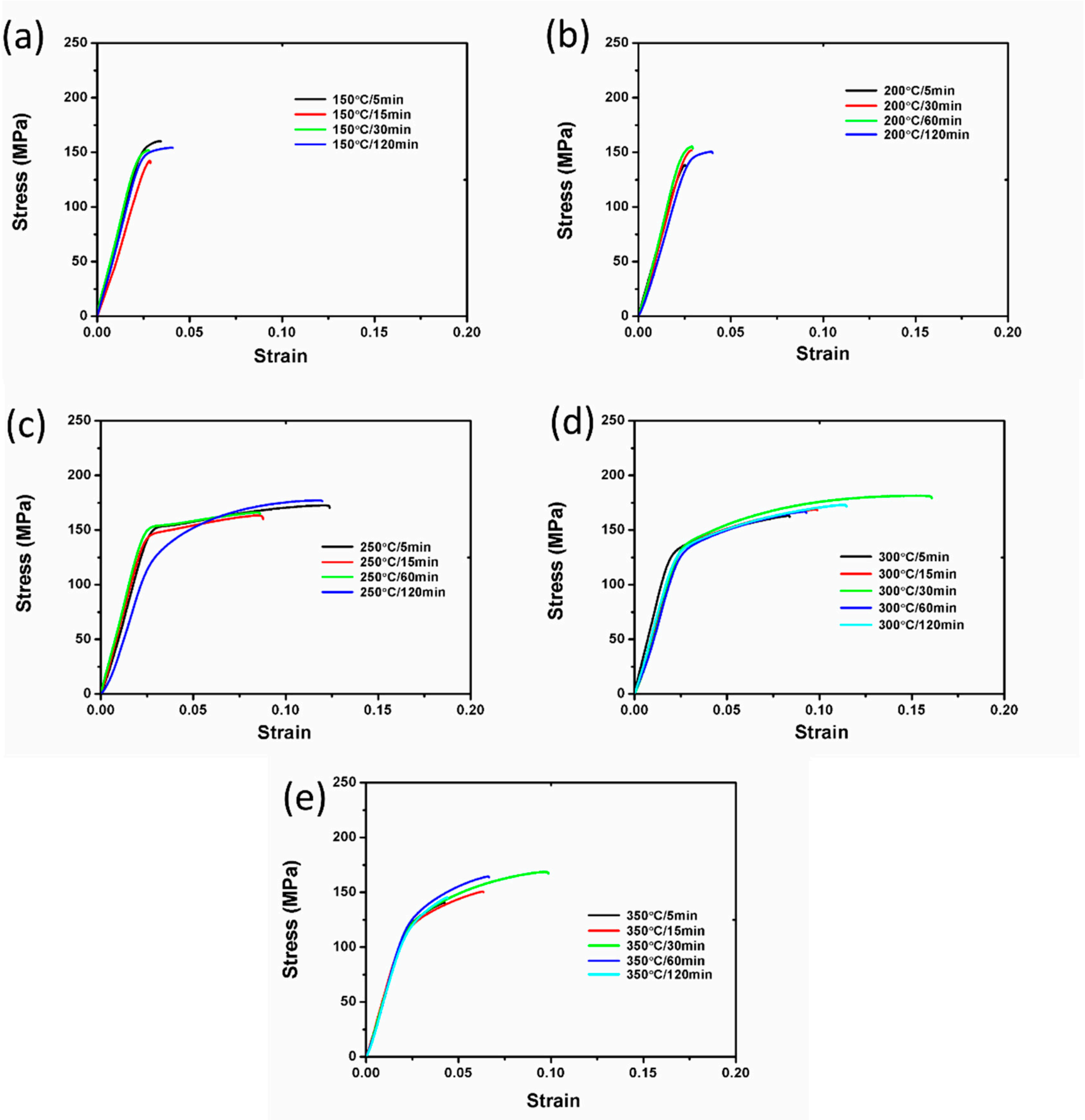

Figure S1. Strain-stress curves of Mg wires annealed at different temperature: (**a**) 150 °C; (**b**) 200 °C; (**c**) 250 °C; (**d**) 300 °C; (**e**) 350 °C.

The mechanical properties of thinner annealed Mg wires were also tested. The results are listed in Figure S2 and Table S1. The tested wires were firstly cold drawn with accumulated true strain of 138 %, and then annealed at 250 °C/5 min, 300 °C/30 min and 350 °C/120 min. Due to the different diameters, the stress and strain values of the thinner (0.2 mm in diameter) Mg wires were different from that of the thick wires to some extent. The thin wires annealed at 300 °C for 30 min exhibited preferable integrated mechanical properties, especially the excellent plasticity. The results further confirm that annealing treatment at 300 °C/30 min is appropriate for the improvement of mechanical properties of cold drawn Mg wires.

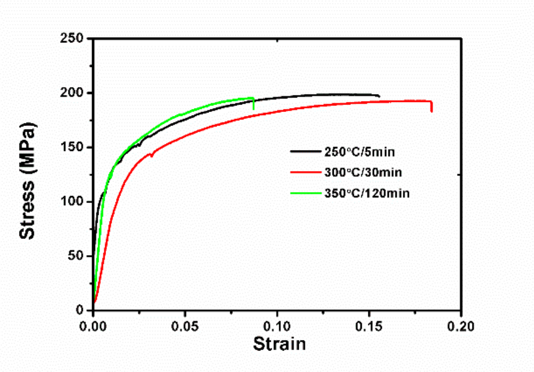

Figure S2. Strain-stress curves of thin Mg wires (0.2 mm in diameter) with true strain of 138% after different annealing treatments.

Table S1. The statistic results of tensile test for thin Mg wires with diameter of 0.2 mm after different annealing treatments.

Annealing process	Ultimate strength(MPa)	Yield strength (MPa)	Elongation(%)
250 °C /5 min	199	100	13.3
300 °C/30 min	196	96	16.1
350 °C/120 min	195	92	9.1

The metallographic microstructures of Mg wires with the diameter of 1.5 mm annealed at 150 °C, 200 °C, 250 °C, 300 °C and 350 °C for 15 min and 60 min are shown in Figure S3.

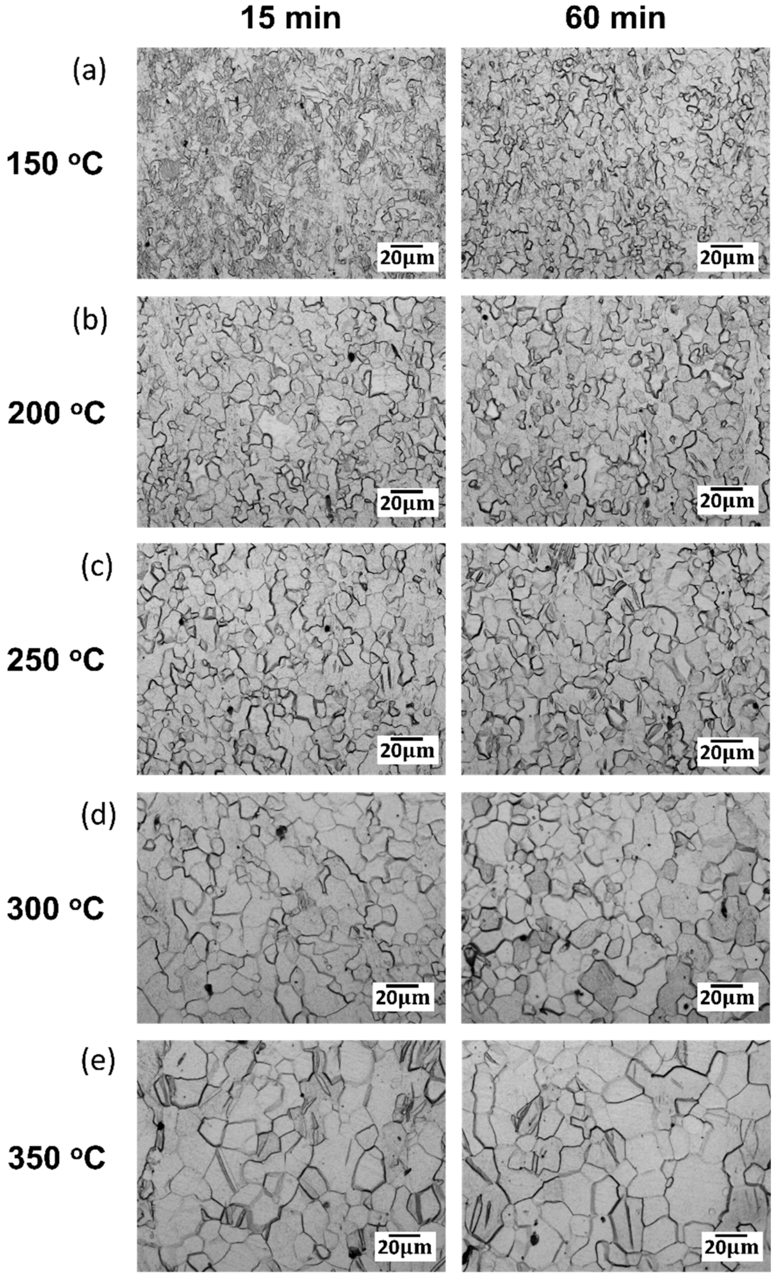

Figure S3. Annealed microstructures of pure Mg with different annealing process. (**a**) 150 °C; (**b**) 200 °C; (**c**) 250 °C; (**d**) 300 °C; (**e**) 350 °C.
